# Early Acute Aortic Dissection After Coronary Artery Bypass Grafting

**DOI:** 10.21470/1678-9741-2023-0342

**Published:** 2025-02-05

**Authors:** Živojin S. Jonjev, Adam Adam, Novica Kalinić, Tamaš Vaštag, Ilija Bjeljac

**Affiliations:** 1 Institute for Cardiovascular Diseases of Vojvodina, Clinic of Cardiovascular Surgery, Sremska Kamenica, Serbia; 2 Faculty of Medicine, University of Banja Luka, Banja Luka, Republic of Srpska, Bosnia & Herzegovina; 3 John H. Stroger, Jr. Hospital of Cook County, Chicago, Illinois, Unites States of America

**Keywords:** Coronary Artery Bypass, Aortic Dissection, Cardiac Surgical Procedures, Thoracic Aorta, Aortic Dissection

## Abstract

Patients having Stanford type A acute dissection soon after cardiac surgery have a high risk of rupture and death. The presentation, management, and outcome of primary dissection of the ascending aorta (Stanford type A or De Bakey type I or II) are well described. However, patients with Stanford type A acute aortic dissection soon (3-4 weeks) after primary cardiac surgery have distinctly different presentation, management, and postoperative outcome. In this report, we describe the clinical and surgical findings of a patient with early Stanford type A acute aortic dissection four weeks after primary coronary artery bypass grafting.

## INTRODUCTION

The overall mortality rate after coronary artery bypass grafting (CABG) is < 1% with various causes of death. Early acute aortic dissection soon after CABG has been recognized as a complication with the most extensive mortality rate, and if untreated could reach mortality of almost 100%. Most of the cases are diagnosed postmortem, with an incidence of 0.03-0.05% at autopsy^[1]^. However, improved imaging modalities and routine echocardiography examinations demonstrated that the true incidence of these findings is underestimated. In this report, we present a case of a man who had Stanford type A acute aortic dissection four weeks after CABG.

### Clinical Summary

A 62-year-old Caucasian man with low social profile was transferred from an outside hospital with a sign of unstable angina. Prior to admission, the patient had episode of heart decompensation predominantly related with left heart failure. At the time of admission, chest roentgenogram demonstrated normal size of the heart, and electrocardiogram confirmed ST segment depression (-2 mm) in left precordial channels (V3-V6), sinus tachycardia (heart rate [HR] = 107/min), and occasional ventricular ectopic contractions. The preoperative transthoracic echocardiography (TTE) showed preserved left ventricular systolic function (ejection fraction = 61%), normal aortic valve, and marginal enlarged diameter of the ascending aorta (≈ 4.5 cm). Sixty-four multislice computerized tomography (MSCT) of the chest confirmed dilation of the ascending aorta with diameter of 4.8 cm. Coronary angiography showed significant (> 90%) triple vessel coronary artery disease, and the patient was selected for elective coronary artery bypass grafting.

The aorta was cross-clamped, and the heart was arrested by anterograde administration of cold, crystalloid St. Thomas #2 cardioplegia, directly applied into the ascending aorta. Standard on-pump procedure with single venous cannulation was carried out. Left anterior descending artery (LAD) was grafted with skeletonized left internal mammary artery (LIMA)^[2]^, and right coronary artery (RCA) and circumflex artery (Cx) were grafted with saphenous vein grafts (SVG). The postoperative course of the patient was uneventful, and the patient was discharged on the postoperative day (POD) #7 without complication. Five days after discharge, the patient was starting to have shortness of breath and fatigue, and two weeks later he was readmitted to the hospital. At the time of readmission, the patient was dehydrated, with atrial fibrillation (HR = 145/min) and low blood pressure (90/55 mmHg). TTE showed significant pericardial effusion (> 2.5 cm, “swinging heart”), and the patient was scheduled for elective pericardial drainage. Intraoperative transesophageal echocardiography showed small coagulum behind the right atrium, and normal postoperative finding after procedure.

**Fig. 1:**
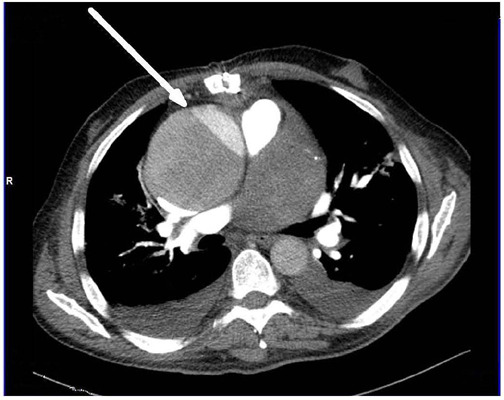
Preoperative 64 multislice computed tomography of the chest. Arrow points acute aortic dissection (Stanford A).

**Fig. 2:**
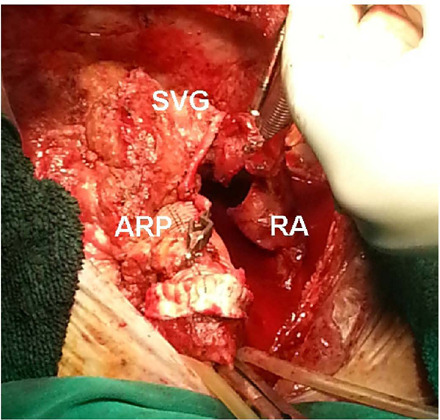
Gross view of the dissecting aorta after graft implantation. RA=right atrium; ARP=aortic root prosthesis; SVG=saphenous vein graft for the right coronary artery.

Four weeks after CABG and one week after pericardial drainage, the patient was weak, without significant clinical improvement. MSCT of the chest documented multi fragmentation of the sternal bone with normal ascending aorta, and patient was scheduled for sternal re-closure, electively. Three-day later control MSCT of the chest revealed dissection of the aorta (Stanford type A) with suspected rupture into the right atrium (Figure 1). The patient was urgently reoperated on. A modified Bentall-DeBono procedure was performed with implantation of the #23 St Jude composite, mechanical prosthesis (Abbott Inc., Abbott Park, ILL). Previously implanted SVG for RCA and Cx were reattached into aortic prosthesis, and the right atrium was directly sutured with 5/0 continuous polypropylene suture (Figure 2, Video 1). The patient cross-clamping time was 192 minutes, and he was weaned off cardiopulmonary bypass after 240 minutes. He had an uneventful postoperative course and was discharged on POD #12.

**Video 1:**
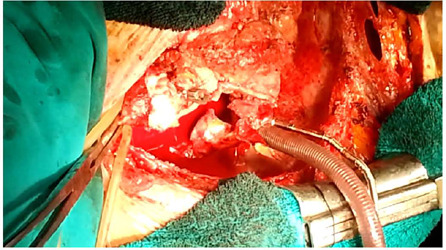
Intraoperative view of the dissecting aorta. Link: https://youtu.be/kGZdEHxWnBI

## QUESTIONS

**A.** What are the predisposing factors associated with the risk of developing aortic dissection soon after previous cardiac surgery?
**B.** Is the clinical presentation of such phenomena standardized?
**C.** What are the strategies for further management of the aortic dissection soon after previous cardiac surgery?

### Discussion of Questions

**Question A.** Acute aortic dissection is a rare complication soon (3-4 weeks) after CABG. Historically, it was an incidental autopsy finding with undetermined clinical significance. The mechanism of delayed aortic dissection after CABG is still under investigation. In most of the cases, aortic dissection after CABG is iatrogenic (*e.g.*, site of aortic cannulation, site of proximal anastomosis, site of cardioplegia cannula, side biting aortic clamp). Nevertheless, there was an opinion that such catastrophic complication could be related to the underline predisposing factors especially in fragile patients with dilated aorta (*e.g.*, arterial hypertension, aortic wall weakness, atherosclerosis) rather than technical surgical errors and cross-clamping of the aorta^[1,3,4]^. In our case, the aorta was totally cross-clamped, without usage of side aortic clamp, and the possible entry site of dissection was 2 cm above the left coronary ostia. That was far away from surgical manipulation; therefore, we speculated that the reason of aortic dissection could be increased wall stress caused by all previously mentioned predisposing factors.
**Question B.** Early acute type A dissection after CABG has different clinical presentation, from totally asymptomatic to highly unstable patients depending of the mechanism of dissection and the heart structures involved^[5,6]^. In the case of severe and extensive aortic damage, malignant arrhythmias, syncope, cardiac arrest, and death were seen. On the contrary, minor, localized aortic tear in most cases remains unrecognized for many years because of the preservation of the vital heart structure which keeps stability in cardiovascular hemodynamic. Thus, cardiac tamponade and free rupture occur rarely, probably due to additional support of the adjacent local adhesions^[6]^.
**Question C.** Even with modern echocardiography, such injuries are difficult to detect. If TTE is normal, MSCT should be performed in symptomatic patients to confirm better visualization of the aorta^[5,6]^.

The surgical treatment depends on the severity and variety of the local finding. Hemodynamic instability is such cases are rare. Thus, favorable hemodynamic status and standardized management result in acceptable low operative mortality^[1,5,6]^. Special care should be taken during resternotomy since the risks of cardiac injury and catastrophic hemorrhage are increased. In our case, a modified Bentall-DeBono procedure was performed. We used composite mechanical prosthesis to reconstruct aortic root. Previously implanted skeletonized LIMA attached to the LAD was preserved, and SVG for RCA and Cx were reattached into woven base aortic prosthesis. Reconstruction of the right atrium was also challenging due to adjacent huge hematoma and dissecting aneurysm.

## LEARNING POINTS

The numbers of cardiac surgery procedures increase tremendously every year, and it is important to know that acute aortic dissection soon after cardiac surgery procedure is possible. Most of the publications including the actual guidelines emphasize that the traditionally accepted factors for ascending aorta evaluation are under a great debate. It seems that the additional factors, not just aortic diameter and the rate of enlargement, should lead to consideration of individual preventive aortic replacement even when the generally accepted diameter of 5 cm has not yet been reached. A lower threshold should be considered in patients with additional risk factors. Therefore, we report a very rare case of early acute aortic dissection after CABG with potential implications for management. In our hands, urgent surgical repair of the aortic root followed with reattachment of the SVG into aortic prosthesis had a favorable outcome. The patient survived the reoperation and stay well and without recurrence a year after surgery.

## Abbreviations, Acronyms & Symbols

ARP: Aortic root prosthesis
CABG: Coronary artery bypass grafting
Cx: Circumflex artery
HR: Heart rate
LAD: Left anterior descending artery
LIMA: Left internal mammary artery
MSCT: Multislice computerized tomography
POD: Postoperative day
RA: Right atrium
RCA: Right coronary artery
SVG: Saphenous vein grafts
TTE: Transthoracic echocardiography
